# Supplementary material for: Functional and structural characterization of interactions between opposite subunits in HCN pacemaker channels
Source: Commun Biol. 2022 May 9;5:430. doi: 10.1038/s42003-022-03360-6 (PMC9085832; doi:10.1038/s42003-022-03360-6)
Supplement: Supplementary file 2 — Supplementary Information [file 42003_2022_3360_MOESM2_ESM.pdf]

Supporting Information

Functional and structural characterization of interactions between opposite subunits in HCN channels

Mahesh Kondapuram[1#], Benedikt Frieg[2#], Sezin Yüksel[1], Tina Schwabe[1], Christian Sattler[1], Marco Lelle[1], Andrea Schweinitz[1], Ralf Schmauder[1], Klaus Benndorf[1], Holger Gohlke[2,3,4*], and Jana Kusch[1*]

[1]Universitätsklinikum Jena, Institut für Physiologie II, Jena, Germany
[2] John von Neumann Institute for Computing (NIC), Jülich Supercomputing Centre (JSC), and Institute of Biological Information Processing (IBI-7: Structural Biochemistry), Forschungszentrum Jülich GmbH, Jülich, Germany.
[3] Institut für Pharmazeutische und Medizinische Chemie, Heinrich-Heine Universität Düsseldorf, Düsseldorf, Germany.
[4] Institute of Bio- and Geosciences (IBG-4: Bioinformatics), Forschungszentrum Jülich GmbH, Jülich, Germany.

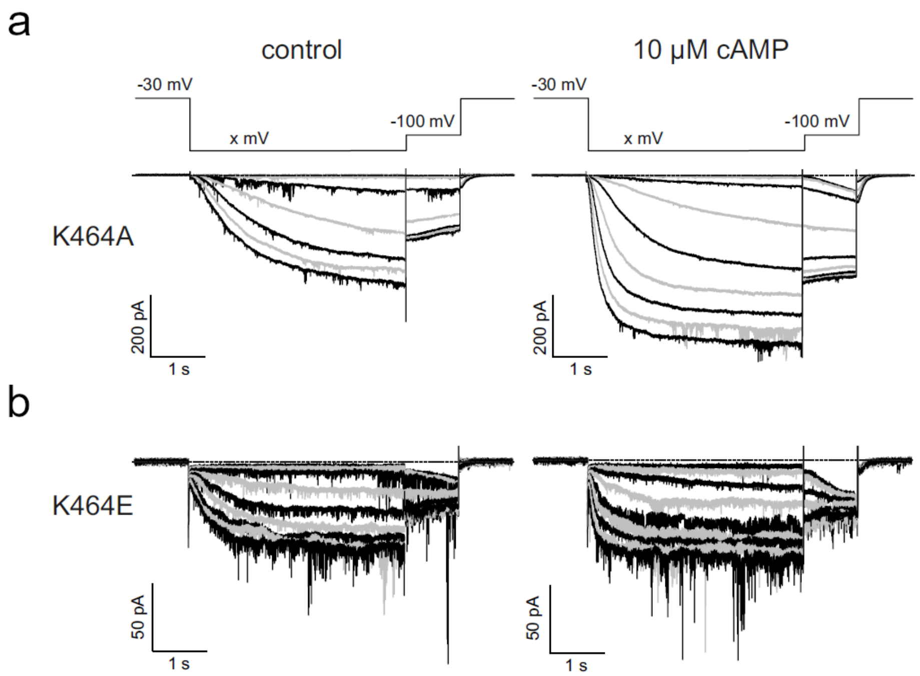

Figure S1: Voltage-dependent activation of K464 mutants at zero and saturating [cAMP] (10 µM).

Representative current traces for K464A (**a**) and K464E (**b**). The voltage protocol is illustrated above. Command voltages were applied in 10 mV-increments ranging from -70 mV to -150 mV.

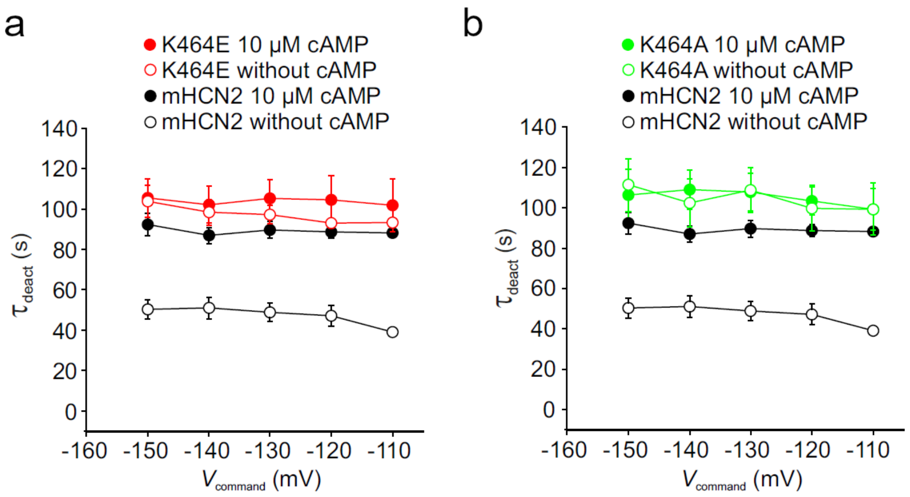

Figure S2: Deactivation kinetics for K464 mutants.

Deactivation time constants at zero and saturating [cAMP] for K464E (**a**) and K464A (**b**) in comparison to mHCN2 (n = 5 to 7).

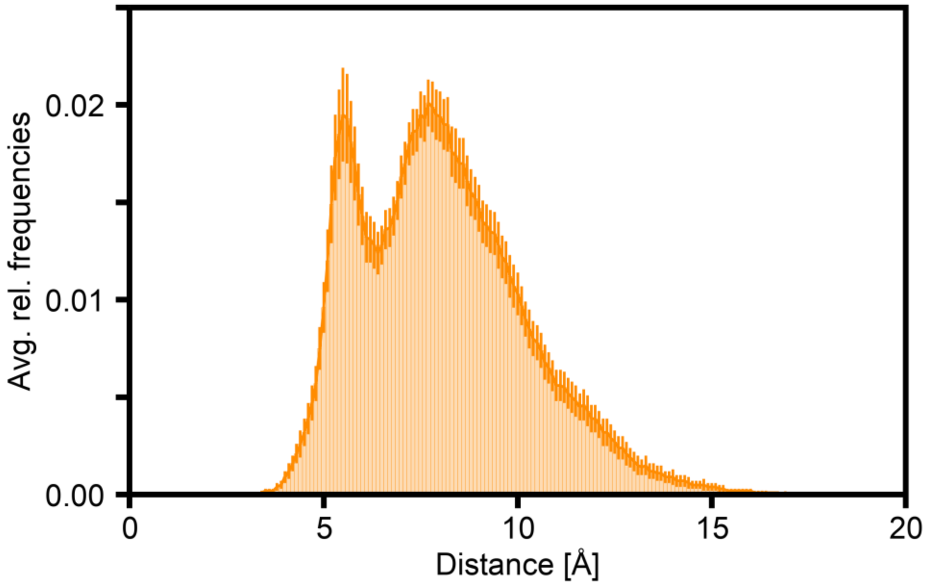

Figure S3: Distance measurements between K464E and M155.

Histogram (bin size 0.1 Å) of the minimal distance between the side-chain oxygen atom in residue K464E and any backbone atom in residue M155. The histogram is normalized to the sum of all bins. The average values were calculated over all four subunits of the mHCN2 channel and over 20 independent replicas (n = 80). The error bars denote the standard error of the mean.

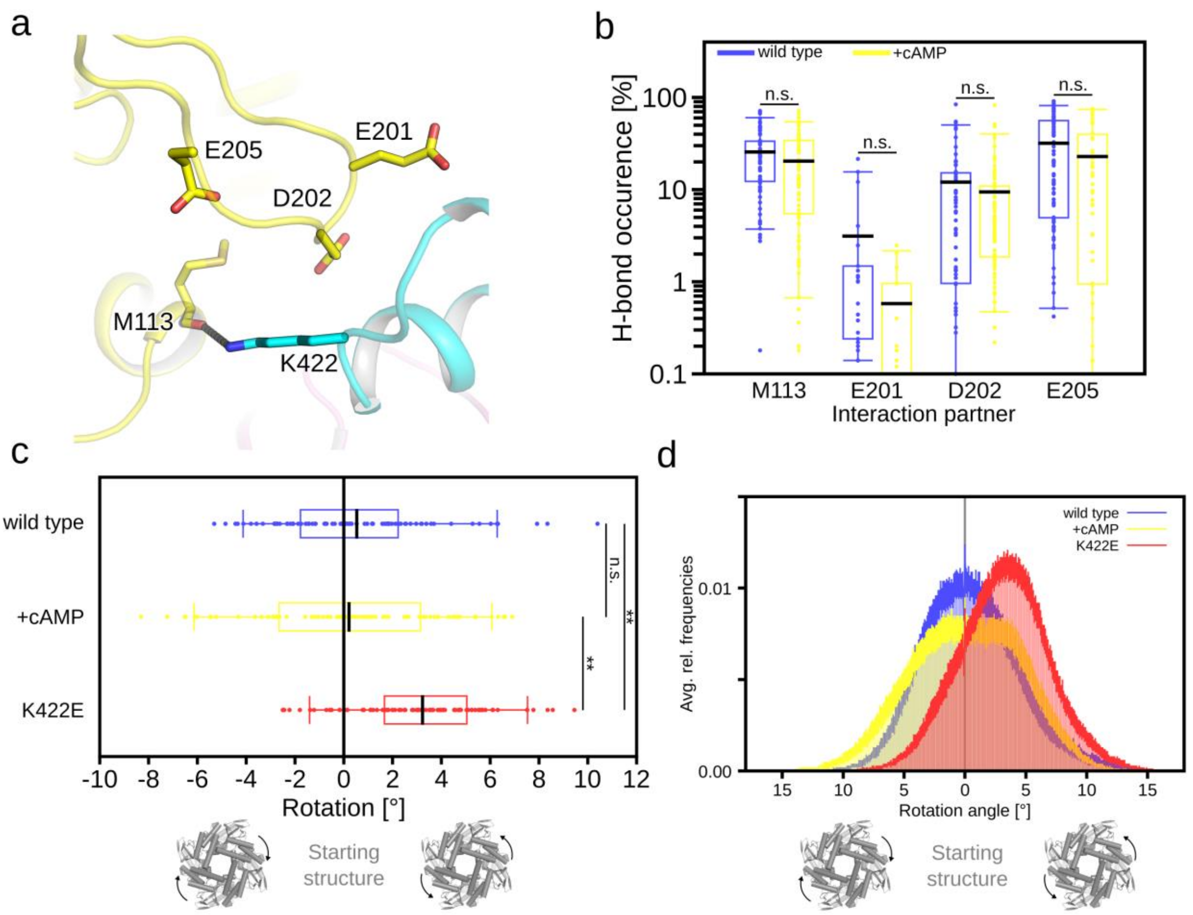

Figure S4: Analyses of MD simulations of the wild type and K422E hHCN1 channel.

a: A close-up view of the interaction site between two opposite subunits (colored cyan and yellow) in the hHCN1 channel (residues M94 to D608; based on PDB ID 5U6O[1] (used for MD simulations). M113, E201, D202, E205, and K422 (homologous residues to M155, E243, D244, E247, and K464 in the mHCN2 model) are depicted as sticks. The interaction between K422 and M113 is depicted as a gray dotted line. **b**: Average occurrence frequency of hydrogen bond interactions between two opposite subunits involving K422. K422 resides on subunit *i*, and the interaction partners M113, E201, D202, and E205 reside on subunit *i* + 2. As to M113, we only considered the backbone oxygen as H-bond acceptor; for E201, D202, and E205, we only considered the side-chain oxygen atoms, as we considered these interactions more favorable compared to backbone interactions. **c**: Average rotation angle relative to the channel pore in the starting structure. **d**: Average histogram (bin size 0.1°) of the rotation angle relative to the channel pore normalized to the apo wild type average (the vertical line at 0°). In **c**+**d**, the direction of rotation is visualized by the scheme below the panels. In **b**+**c**, the average values (black lines) were calculated individually for each of the four subunits and throughout 20 independent MD simulation replicas (n = 80) with the individual data points shown as circles. The boxes denote the range from the 25th to 75th percentile and include 50% of the data points. The whiskers denote the 5th to 95th percentile and include 90% of the data points.

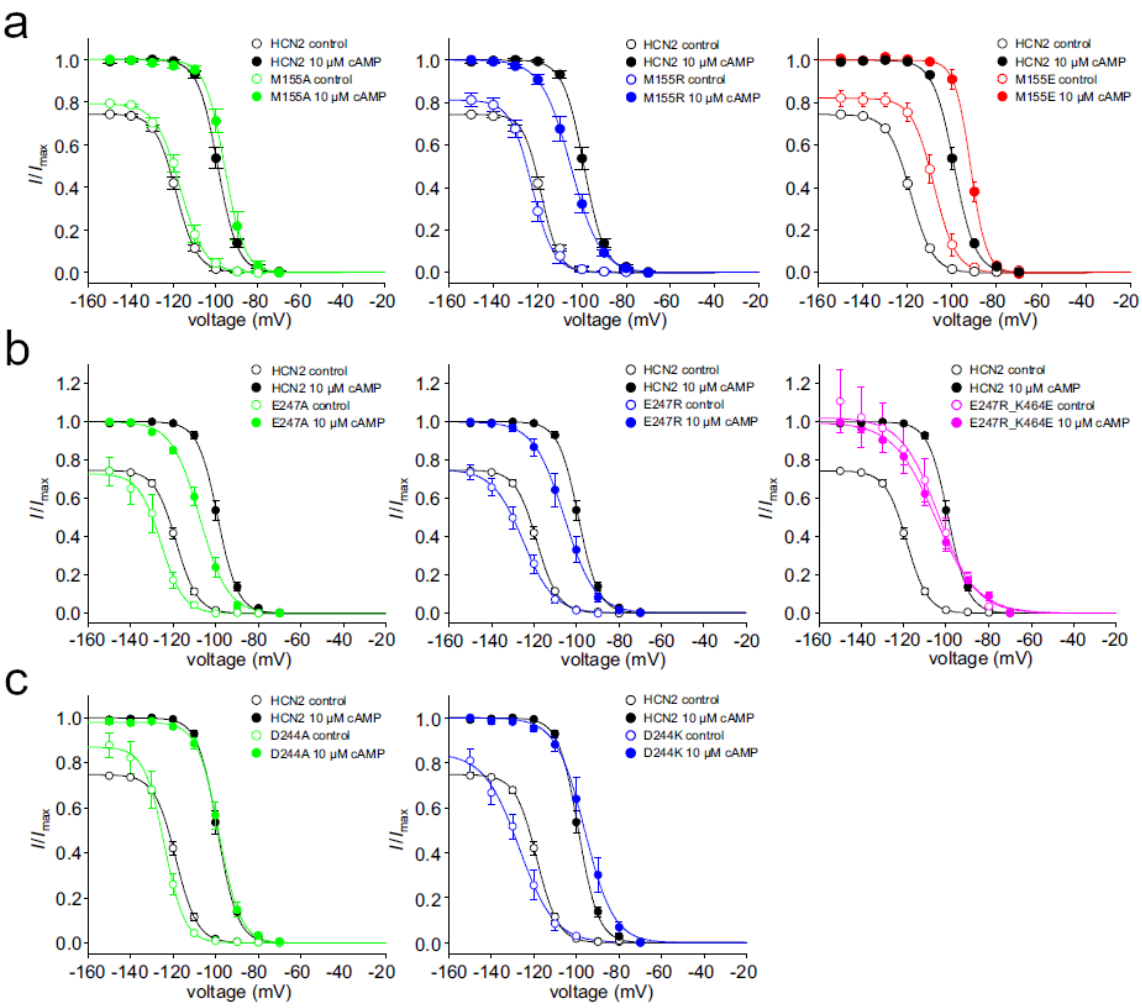

Figure S5: Steady-state activation relationships for mutated constructs in comparison with mHCN2 wildtype.

Shown are steady-state activation relationships for **a**) M155A, M155R, M155E, **b**) E247A, E237R, E247R_K464E, **c**) D244A, D244K in the absence and presence of saturating [cAMP] (colored symbols as incidicated). In each case, open and filled symbols represent mHCN2 in the absence and presence of saturating [cAMP], respectively. The Boltzman equation was fitted to the data yielding $V_{1/2}$ and $z\delta$.

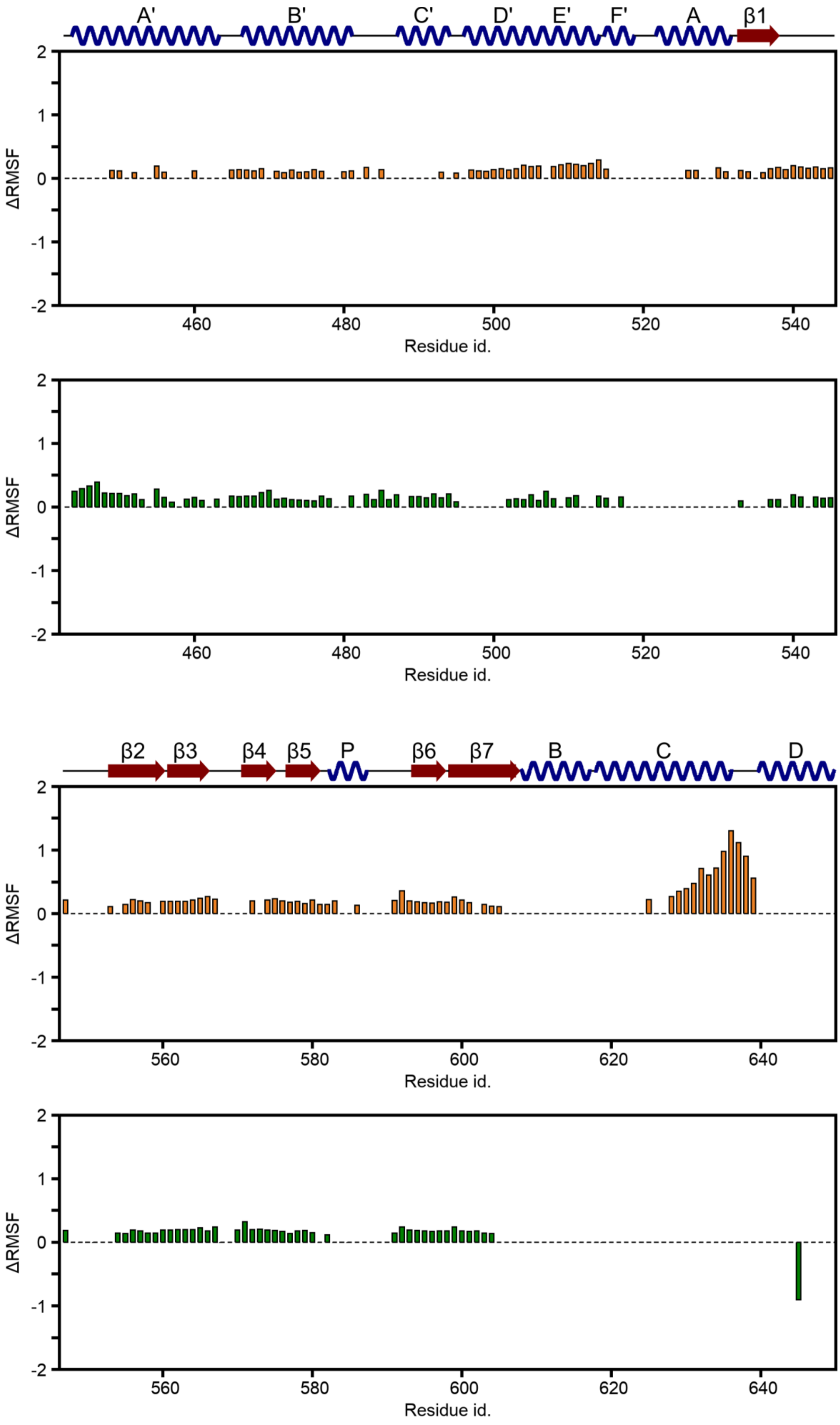

A'
B'
C'
D'
E'
F'
A
β1
β2
β3
β4
β5
P
β6
β7
B
C
D
ΔRMSF
Residue id.
2
1
0
-1
-2
460
480
500
520
540
560
580
600
620
640

Figure S6: Changes in side-chain mobility within the CL-CNBD induced by cAMP binding or K464E substitution.

The bar plots show the residue-wise average ΔRMSF (root mean square fluctuations; see also eq. (4); n = 80 independent replicas)). The orange bars show the ΔRMSF for the cAMP-bound wild type channel, and the green bars the ΔRMSF for the *apo* K464E channel, with respect to the *apo* wild type channel. If the residue-wise RMSF is not significantly different from the *apo* wild type channel ($p > 0.05$; p-value by t-test), ΔRMSF was set to zero. The top two panels show the ΔRMSF for residues 443 – 546 and the lower two panels for residues 547 – 650. The secondary structure of the CL-CNBD is schematically visualized, with helices shown as blue springs, β-strands as red arrows, and loops as black lines. The nomenclature was adapted from ref. Lee and MacKinnon (2017)[1].

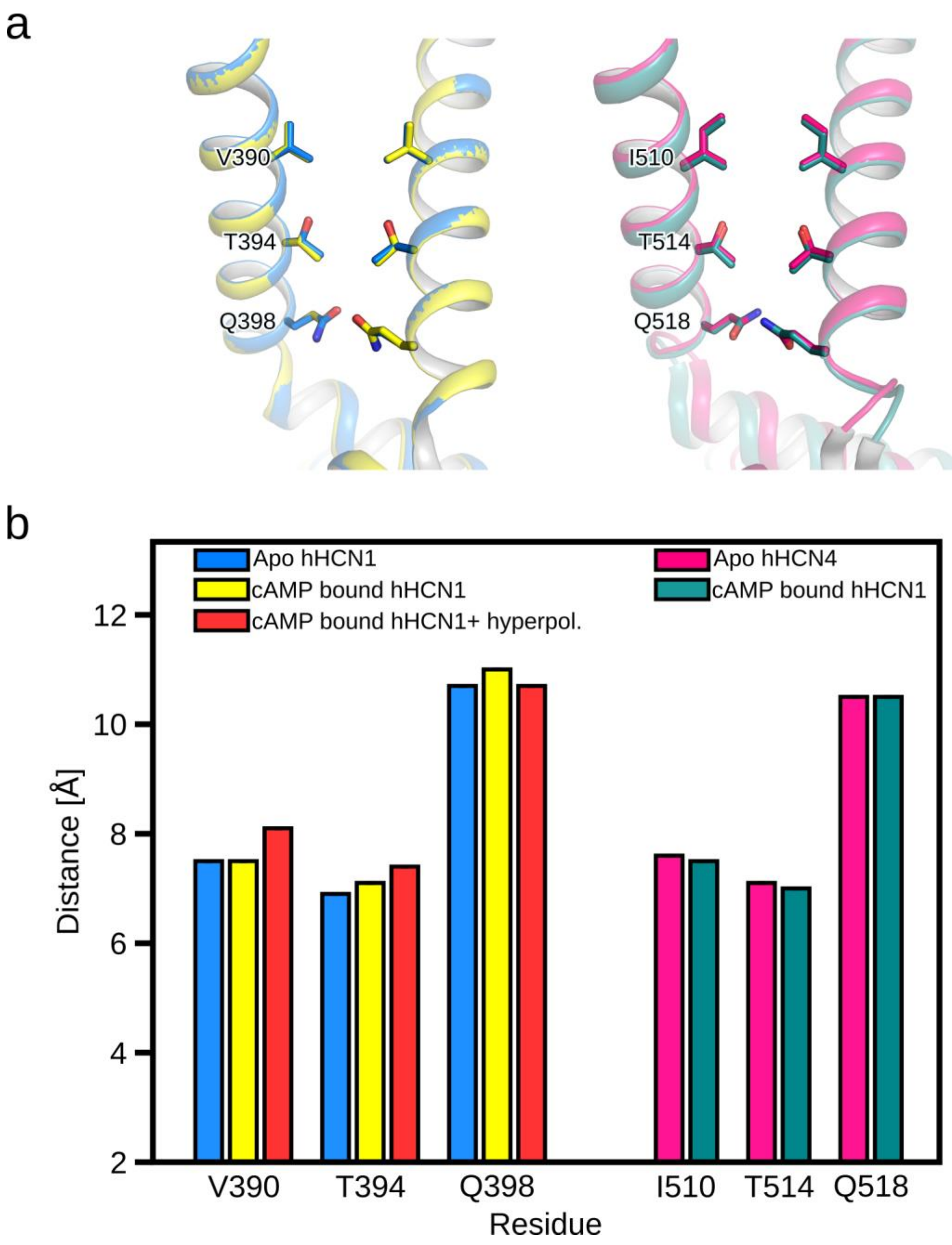

Figure S7: Conformational analyses of the channel gate of experimental structures.

a: The left structure shows the superposition of the *apo* (light blue, PDB ID 5U6O[1] and cAMP-bound (yellow, PDB ID 5U6P[1]) gate region of the hHCN1, and the right panel the *apo* (magenta, PDB ID 6GYN[2] and cAMP-bound (dark cyan, PDB ID 6GYO[3]) hHCN4 channel. The protein structure is shown as cartoon representation with amino acids forming the gate shown as stick models. **b:** Distance measurements between the C_β-atoms of two opposite amino acids that form the gate. The bar plot depicts single measures based on the available 3D structures[1-3].

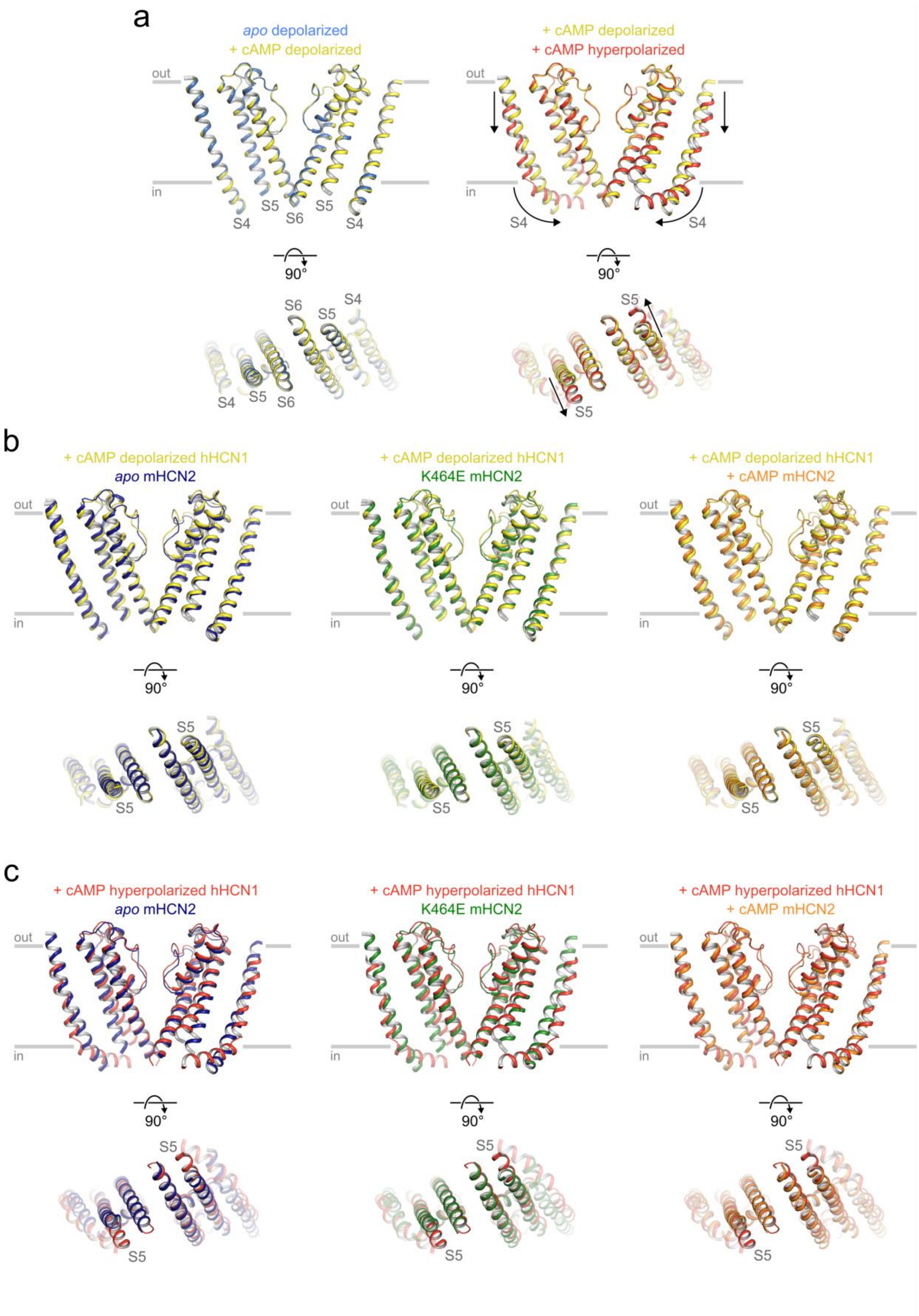

Figure S8: Comparative analyses of the S4/S5/S6 transmembrane portion in HCN channels.

a: Superposition of the S4/S5/S6 transmembrane portion of the hHCN1 with only two opposite subunits shown. The left panel shows the superposition of the apo (blue) and cAMP bound (yellow) structures in the depolarized conformation[1]. The right panel shows the superposition of the depolarized (yellow) and hyperpolarized (red)

conformations bound to cAMP[1,4]. In the right panel, structural changes induced by hyperpolarization (lowering and bending of the S4 helix and movement of the S5 helix) are visualized by arrows. In the lower panels, the channels are rotated by 90°, such that the intracellular portion is now oriented towards the reader. The gray labels depict relevant structural domains (the S4, S5, and S6 helices). The gray bars depict the approximate location of the membrane bilayer. The layout is the same in panels **b** and **c**. **b, c:** From left to right, the panels show the superposition of the average apo wild type mHCN2 (dark blue), the average K464E mHCN2 (green), and the average cAMP bound wild type mHCN2 (orange) channels from MD simulations onto the cAMP-bound and depolarized conformation (**b**) or cAMP-bound and hyperpolarized conformation (**c**) of hHCN1 [4].

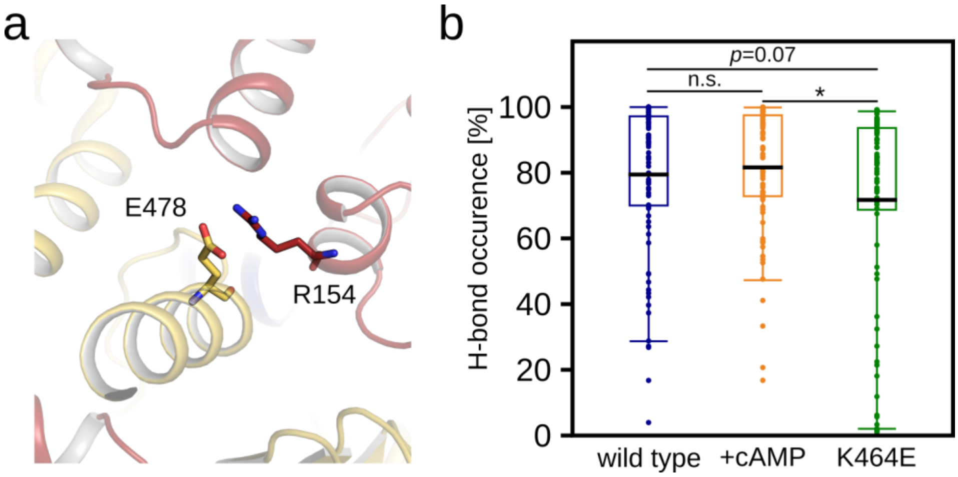

Figure S9: Interaction between C-linker and HCN domain of two neighboring subunits.

a: A close-up view shows the interaction between R154 (HCN domain on subunit *i*) and E478 (C-linker on subunit *i* + 1). The two neighboring subunits are colored differently. **b**: Average occurrence frequency of hydrogen bond interactions between R154 and E478 from panel **c**. The average values (black lines) were calculated individually for each of the four subunits and throughout 20 independent MD simulation replica (n = 80) with the individual data points shown as circles. The boxes denote the range from the 25th to 75th percentile and include 50% of the data points. The whiskers denote the 5th to 95th percentile and include 90% of the data points. (p-value by t-test; * $p < 0.05$; n.s. not significantly different).

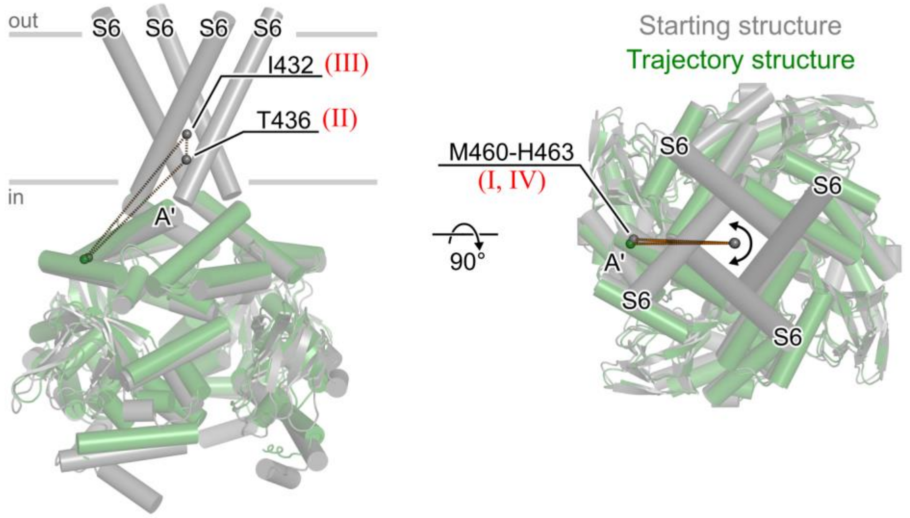

Figure S10: Definition of the rotation angle measurements.

To investigate the rotation of the CL-CNBD relative to the channel pore of the starting structure, we measured the dihedral angle defined by the four reference points **I** – **IV** (red labels): **I**) The center of mass (COM) of C_{α}-atoms of the four-terminal residues (M460-H463) of the A'-helix of the C-linker of the starting structure (gray cartoon representation), **II**) the COM of C_{α}-atoms of T436 on the S6 helix of each subunit of the starting structure, **III**) the COM of C_{α}-atoms of I432 on the S6 helix of each subunit of the starting structure, and **IV**) the COM of C_{α}-atoms of the four-terminal residues (M460-H463) of the A'-helix of the C-linker, but now considering the atoms of structures throughout the MD trajectory (green cartoon representation). The COMs of **I** – **IV** are depicted as spheres, and the dihedral is schematically represented by dashed lines. The left structure panel shows the channel from the side, the right from the top (the extracellular domains are now oriented towards the viewer). The gray bars depict the approximate location of the membrane bilayer. Only the S6 helices of the channel pore are shown for clarity. Helical structures are shown as cylinders.

SUPPLEMENTARY REFERENCES

1 Lee, C. H. & MacKinnon, R. Structures of the Human HCN1 Hyperpolarization-Activated Channel. *Cell* **168**, 111-120 e111, doi:10.1016/j.cell.2016.12.023 (2017).

2 Shintre, C. A., Pike, A.C.W., Tessitore, A., Young, M., Bushell, S.R., Strain-Damerell, C., Mukhopadhyay, S., Burgess-Brown, N.A., Huiskonen, J.T., Arrowsmith, C.H., Edwards, A.M., Bountra, C., Carpenter, E.P. (The Protein Data Bank 2019).

3 Shintre, C. A., Pike, A.C.W., Tessitore, A., Young, M., Bushell, S.R., Strain-Damerell, C., Mukhopadhyay, S., Burgess-Brown, N.A., Huiskonen, J.T., Arrowsmith, C.H., Edwards, A.M., Bountra, C., Carpenter, E.P., Structural Genomics Consortium (SGC). (Protein Data Base, 2019).

4 Lee, C. H. & MacKinnon, R. Voltage Sensor Movements during Hyperpolarization in the HCN Channel. *Cell* **179**, 1582-1589 e1587, doi:10.1016/j.cell.2019.11.006 (2019).
