# Supplementary material for: Functional and structural characterization of interactions between opposite subunits in HCN pacemaker channels
Source: Commun Biol. 2022 May 9;5:430. doi: 10.1038/s42003-022-03360-6 (PMC9085832; doi:10.1038/s42003-022-03360-6)
Supplement: Supplementary file 3 — Reporting Summary [file 42003_2022_3360_MOESM3_ESM.pdf]

nature research

Corresponding author(s): Jana Kusch, Holger Gohlke

Last updated by author(s): Feb 28, 2022

Reporting Summary

Nature Research wishes to improve the reproducibility of the work that we publish. This form provides structure for consistency and transparency in reporting. For further information on Nature Research policies, see our Editorial Policies and the Editorial Policy Checklist.

Statistics

For all statistical analyses, confirm that the following items are present in the figure legend, table legend, main text, or Methods section.

n/a	Confirmed	
☐	☒	The exact sample size (*n*) for each experimental group/condition, given as a discrete number and unit of measurement
☐	☒	A statement on whether measurements were taken from distinct samples or whether the same sample was measured repeatedly
☐	☒	The statistical test(s) used AND whether they are one- or two-sided<br>*Only common tests should be described solely by name; describe more complex techniques in the Methods section.*
☒	☐	A description of all covariates tested
☒	☐	A description of any assumptions or corrections, such as tests of normality and adjustment for multiple comparisons
☒	☐	A full description of the statistical parameters including central tendency (e.g. means) or other basic estimates (e.g. regression coefficient) AND variation (e.g. standard deviation) or associated estimates of uncertainty (e.g. confidence intervals)
☒	☐	For null hypothesis testing, the test statistic (e.g. *F*, *t*, *r*) with confidence intervals, effect sizes, degrees of freedom and *P* value noted<br>*Give P values as exact values whenever suitable.*
☒	☐	For Bayesian analysis, information on the choice of priors and Markov chain Monte Carlo settings
☒	☐	For hierarchical and complex designs, identification of the appropriate level for tests and full reporting of outcomes
☒	☐	Estimates of effect sizes (e.g. Cohen's *d*, Pearson's *r*), indicating how they were calculated

Our web collection on statistics for biologists contains articles on many of the points above.

Software and code

Policy information about availability of computer code

Data collection	Patch-Clamp-Data were collected using PatchMaster (Harvard Apparatus, Holliston, US). PCF-data were collected using ZEN (Zeiss, Germany) and ISO3 (MFK, Germany).
Data analysis	For analysing electrophysiological and PCF data OriginPro9.0G (Northampton, USA) was used.

For manuscripts utilizing custom algorithms or software that are central to the research but not yet described in published literature, software must be made available to editors and reviewers. We strongly encourage code deposition in a community repository (e.g. GitHub). See the Nature Research guidelines for submitting code & software for further information.

Data

Policy information about availability of data

All manuscripts must include a data availability statement. This statement should provide the following information, where applicable:

- Accession codes, unique identifiers, or web links for publicly available datasets
- A list of figures that have associated raw data
- A description of any restrictions on data availability

Data that support the findings of this study are available on reasonable request.

Field-specific reporting

Please select the one below that is the best fit for your research. If you are not sure, read the appropriate sections before making your selection.

☒ Life sciences ☐ Behavioural & social sciences ☐ Ecological, evolutionary & environmental sciences

For a reference copy of the document with all sections, see nature.com/documents/nr-reporting-summary-flat.pdf

Life sciences study design

All studies must disclose on these points even when the disclosure is negative.

Sample size	Experimental part: There was no sample-size calculation performed for electrophysiological or microscopic recordings. At least 3 recordings for the PCF approach and at least 5 recordings for the patch-clamp approach were performed. This number was increased when a high variablility within the first set of recordings was observed.<br><br>Computational part: We performed 20 independent NPT production simulations. As the initial velocities were randomly assigned during the first step of the NPT production simulation, each production simulation can be considered as an independent replica.
Data exclusions	No data were excluded.
Replication	Experimental part: Both patch-clamp and PCF recordings have been performed under the same technical conditions by two independent co-authors. Data sets of author 1 could be confirmed by author 2.<br><br>Computational part: To derive independet replicas, the initial velocities were randomly assigned during the first step of the NPT production simulation. The applied random seed for every production simulation, however, is well documented and will be available from the corresponding author on reasonable request.
Randomization	Experimental part: For all patch-clamp and PCF recordings Xenopus laevis oocytes were harvested in our lab from femal animals or purchased as ready to use oocytes. Each batch of oocytes was used for several types of RNA to assure most similar conditions for the different mutations and to measure them in parallel during the same time window.
Blinding	Experimental part: Investigators were not blinded, because different HCN mutations required different recording conditions, mainly regarding the concentrations of cyclic nucleotides used. Particularly, for the cost intensive PCF recordings using fluorophore-coupled cAMP, blinding was not possible.

Reporting for specific materials, systems and methods

We require information from authors about some types of materials, experimental systems and methods used in many studies. Here, indicate whether each material, system or method listed is relevant to your study. If you are not sure if a list item applies to your research, read the appropriate section before selecting a response.

Materials & experimental systems

n/a	Involved in the study
☒	☐ Antibodies
☒	☐ Eukaryotic cell lines
☒	☐ Palaeontology and archaeology
☐	☒ Animals and other organisms
☒	☐ Human research participants
☒	☐ Clinical data
☒	☐ Dual use research of concern

Methods

n/a	Involved in the study
☒	☐ ChIP-seq
☒	☐ Flow cytometry
☒	☐ MRI-based neuroimaging

Animals and other organisms

Policy information about studies involving animals; ARRIVE guidelines recommended for reporting animal research

Laboratory animals	Female African claw frogs (Xenopus laevis) were used to harvest oocytes as heterologous cell system. The frogs were between 2 and 3 years old. They were purchased from Nasco (Fort Atkinson, US).
Wild animals	The study did not involve wild animals.
Field-collected samples	The study did not involve samples collected from the field.
Ethics oversight	The surgery procedures were carried out in accordance with the German Animal Welfare Act with the approval of the Thuringian State Office for Consumer Protection on 30.08.2013 and 09.05.2018.

Note that full information on the approval of the study protocol must also be provided in the manuscript.
